# Resource use among patients with diabetes, diabetic neuropathy, or diabetes with depression

**DOI:** 10.1186/1478-7547-4-18

**Published:** 2006-10-23

**Authors:** Trong K Le, Stephen L Able, Maureen J Lage

**Affiliations:** 1 Eli Lilly and Company, Indianapolis, IN, USA; 2 HealthMetrics Outcomes Research, Groton, CT, USA

## Abstract

**Background:**

Diabetes is often associated with complications and comorbidities. The purpose of this research is to compare medical resources used by patients with the following diagnoses: diabetes mellitus (DM), diabetic neuropathy (DN), and diabetes mellitus combined with comorbid depression (DD).

**Methods:**

Adult patients who were diagnosed with DM, DN, or DD were included in the study. There were 55,972 patients in the DM cohort, 2,146 in the DN, and 2,379 in the DD. P values for comparisons between the three mutually exclusive cohorts were conducted using the Tukey-Kramer method. Cost comparisons among the cohorts were conducted using a stepwise multivariate regression that controlled for patient characteristics and comorbid conditions.

**Results:**

Individuals in the DM or DN cohorts were generally more likely to use antidiabetic medications than patients in the DD group. Those diagnosed with DN or DD generally used more pain medications than individuals in the DM cohort. The DM cohort had significantly lower diabetes-related total medical costs ($1,297 v $5,125, p < 0.0001) and lower total medical costs ($4,819 v $24,765, p < 0.0001) than the DN cohort. The DM cohort also had significantly lower diabetes-related total medical costs ($1,297 v $3,264, p < 0.0001) as well as significantly lower total medical costs ($4,819 v $19,298, p < 0.0001) than the DD cohort.

**Conclusion:**

Results from this study indicated significant differences in demographic characteristics, comorbidities, and medication use among individuals diagnosed with DM, DN, or DD. These differences translated into significant cost differences. Patients diagnosed with DN or DD had higher diabetes-related costs than patients diagnosed with DM.

## Background

Diabetes was the sixth leading cause of death in the U.S. in 2002 [1]. Between 1990 and 2000 the number of people in the U.S. diagnosed with diabetes increased by 49% [2]. An estimated 18.2 million Americans now suffer with the disease, with 13 million diagnosed and 5.2 million undiagnosed [1]. Accompanying this rise in prevalence is a corresponding rise in the costs of managing the illness. For example, the estimated cost of diabetes in the U.S. increased from $98 billion in 1997 [3] to $132 billion in 2002 [4]. These costs represented direct medical expenditures as well as lost productivity. If diabetes prevalence rates were to remain at current levels, the Census Bureau estimates that the number of people diagnosed with diabetes would increase to nearly 14.5 million by 2010 and to 17.4 million by 2020 [5]. The corresponding annual cost of the illness (in 2002 dollars) would be an estimated $156 billion in 2010 and $192 billion in 2020 [6].

Individuals diagnosed with diabetes mellitus [DM] also may suffer from a wide array of physical complications, such as kidney failure, blindness, lower limb amputations, heart disease, and stroke [7]. Diabetic neuropathy is one of the most common long-term complications of diabetes mellitus. Diabetic neuropathy [DN] has the highest morbidity and mortality rates [8] and is associated with a substantial reduction in quality of life [9]. Approximately 10% of patients have neuropathy at the time of diabetes diagnosis [10,11], and that number increases to as much as 50% among patients who have had diabetes for 15 or more years [10,12].

In addition to physical complications associated with diabetes, individuals with diabetes are also at an increased risk for mental health disorders, particularly depression [13,14]. For example, research has shown that individuals diagnosed with type 2 diabetes were 60% more likely to be diagnosed with depression than patients without type 2 diabetes [15]. Similar results were reported among elderly Medicare claimants [16]. A diagnosis of diabetes and comorbid depression [DD] has been shown to be associated with poor metabolic control, reduced adherence to a medication regime, decreased quality of life [17-19], and higher medical costs [16].

Antidepressants are often prescribed for the treatment of DD as well as for the pain that accompanies DN [20]. Alternatively, medications such as narcotics or non-steroidal anti-inflammatory drugs (NSAIDs) may also be prescribed to help alleviate pain associated with DN or DM. Given the high rate of comorbid depression that accompanies a diagnosis of diabetes and the large percentage of diabetic patients who are eventually diagnosed with diabetic neuropathy, the use of such antidepressants and pain medications may add significantly to costs associated with a diagnosis of diabetes.

The purpose of this study was to examine the impact of a serious physical complication (diabetic neuropathy) and a common mental-health related comorbidity (depression) in the diabetes population. To this end, the study compares patients diagnosed with diabetes mellitus (DM), diabetic neuropathy (DN), and diabetes mellitus combined with comorbid depression (DD) to detect patterns and differences in 1) demographic characteristics; 2) the use of antidiabetic medications, antidepressants, and pain medications; and 3) direct medical costs. Findings in this study can therefore be used to help administrators of managed care plans understand resource utilization among a growing sub-group of their population and can also be used for patient-level economic models of diabetes and diabetes-related comorbidities and complications.

## Methods

Data for this study was obtained from the PharMetrics Patient-Centric Database. This Health Insurance Portability and Accountability Act-compliant database integrates enrollment, medical, and prescription claims data. The database is a systematic sample of commercial health plan information obtained from managed care plans throughout the United States that contains information from over 75 different managed care organizations and consists of more than 55 million lives. All data is standardized across different sources and goes through a rigorous data quality review. For this study, we considered data from the time period January 1, 1995 through August 22, 2003.

The diabetes cohort (DM) consisted of individuals who were first diagnosed with diabetes (based upon an ICD-9 code of 250.0x–250.5x and 250.7x–250.9x) or first received an antidiabetic agent during the time period covering July 1, 1995 through August 23, 2002 (e.g. the identification period), with the first such date identified as the index date. The identification period begins six months after the start period of the data used in the analysis and ends one year prior to the end of the data in order to allow for a six month pre-period and twelve month post-period. In addition, in order to be included in the DM cohort, individuals could not have been diagnosed with either depression (based upon an ICD-9 code of 296.2x, 296.3x, 300.4x, 309.0x, 309.1x or 311.xx) or diabetic neuropathy (based upon an ICD-9 code of 205.6x) and to be continuously insured from the beginning of the pre-period through the end of the post-period. There were 55,972 individuals who fit the above criteria. Similarly, for inclusion in the DN cohort (N = 2,146), individuals were first diagnosed with diabetic neuropathy during the identification period, were not diagnosed with depression, and had continuous insurance coverage from six months prior through twelve months post index date.

For inclusion in the DD cohort, individuals had to be diagnosed with both diabetes and depression. Individuals first diagnosed with diabetes or receiving an antidiabetic medication during the index period were required to have a diagnosis of depression in both the pre-period and the post-period. Alternatively, individuals first diagnosed with depression during the index period were required to have a diagnosis of diabetes or receipt of an antidiabetic medication during both the pre-period and the post-period. These criteria were employed in order to insure that the index date represents the date of comorbid diabetes and depression. In addition, individuals in the DD cohort were required to not be diagnosed with diabetic neuropathy and to have continuous insurance coverage from six months pre through twelve months post index date. There were 2,379 individuals in the DD cohort.

The analysis examined differences in patient characteristics, comorbidity profiles, utilization of medications (antidiabetic, antidepressant, and pain-relieving), and direct medical costs across the DM, DN, and DD cohorts. Patients were identified as having a comorbid condition if they were diagnosed with the condition at any time during the six month pre-period. Direct medical costs were calculated as the sum of all payments and were converted to 2003 values using the medical services component of the consumer price index. Diabetes related costs were defined as a payment associated with a diagnosis of diabetes (whether primary or secondary diagnosis) as well as payments for any antidiabetic medication.

Patient characteristics and medication use were examined for both the DN and DD cohorts compared to the DM cohort as well as between the DN and DD cohorts. P values for differences across cohorts were constructed using the Tukey-Kramer method in order to adjust for multiple pairwise testing. To examine costs between cohorts, multivariate ordinary-least-squares stepwise regressions were estimated controlling for patient characteristics and comorbidities diagnosed in the six months prior to the index date. The logs of costs were used as the dependent variable in order to account for the skewed nature of cost data. From these regression results, costs associated with a diagnosis of DD or DN compared to DM were estimated as were costs associated with a diagnosis of DN compared to DD. All analyses were conducted using SAS Version 9.1 [21].

## Results

Patients in the DN cohort were older than in the DM (with a mean age of 51 yrs vs. 47 yrs, p < 0.0001) or the DD cohort (51 yrs vs. 47 yrs, p < 0.0001), while there was no statistical difference in age between the DM and DD cohorts (p = 0.4626). Whereas over half of the DM and DN patients were males (51%), over half of the DD patients (63%) were females. A majority of the patients were diagnosed in either the South or Midwest and were commercially insured or self-insured (see Table 1).

**Table 1:** Patient Characteristics

**Patient Characteristic**	**Diabetes (N = 55,972)**		**Diabetic Neuropathy (N = 2,146)**		**Diabetes and Depression (N = 2,379)**		**P-Values**		
	
	**Mean**	**Std Dev**	**Mean**	**Std Dev**	**Mean**	**Std Dev**	**DM v DN**	**DM v DD**	**DN v DD**
**Mean Age**	46.67	15.53	50.69	13.35	47.05	12.99	*	0.4626	*
									
**Sex**	**N**	**%**	**N**	**%**	**N**	**%**			
Female	27,229	48.65	1,043	48.60	1,501	6.39	0.9991	*	*
Male	28,743	51.35	1,103	51.40	878	36.91	0.9991	*	*
									
**Region**									
East	12,134	21.68	273	12.72	282	11.85	*	*	0.7533
Midwest	17,044	30.45	813	37.88	1,100	46.24	*	*	*
South	21,468	38.35	902	42.03	592	24.88	***	*	*
West	5,362	9.52	158	7.36	405	17.02	***	*	*
									
**Payment Type**									
Commercial	17,820	31.84	670	31.22	875	36.78	0.8196	*	**
Medicaid	1,143	2.04	45	2.10	188	7.90	0.9846	*	*
Medicaid Risk	4,158	7.43	242	11.28	137	5.76	*	***	*
Self-Insured	10,748	19.20	249	11.60	189	7.94	*	*	***
Other	22,103	37.49	940	43.80	990	41.61	**	0.0952	0.2898
									
**Comorbidities**									
Hypertension	13,578	24.27	975	45.43	1,076	48.23	*	*	0.9863
Hyperlipidemia	5,354	9.57	440	20.50	494	20.77	*	*	0.9549
Cardiovascular Disease	4,457	7.96	565	26.33	494	20.77	*	*	*
Ischemic Heart Disease	2,737	4.89	342	15.94	313	13.16	*	*	*
Obesity	1,668	2.98	130	6.06	236	9.92	*	*	*
Retinopathy	910	1.63	299	13.93	168	7.06	*	*	*
Cataracts	840	1.50	119	5.55	109	4.58	*	*	***
Diabetic Retinopathy	787	1.41	281	13.09	150	6.31	*	*	*
Nephropathy	374	0.67	110	5.13	60	2.52	*	*	*
Osteoporosis	372	0.66	43	2.00	46	1.93	*	*	0.9603
Arteriosclerosis	259	0.46	86	4.01	42	1.77	*	*	*
Blindness	45	0.08	8	0.37	7	0.29	*	***	0.6792
Gangrene	37	0.07	32	1.49	8	0.34	*	**	*
Dialysis	15	0.04	8	0.37	3	0.13	*	0.0572	**

*P-value ≤ 0.0001; **P-value ≤ 0.001; ***P-value ≤ 0.05.

P values based on Tukey-Kramer method to adjust for multiple comparisons.

The most common comorbidities in all three cohorts were hypertension, hyperlipidemia, and cardiovascular diseases. DM patients had a significantly lower rate of hypertension, hyperlipidemia, and cardiovascular diseases compared to patients with DN or DD; however, there were no significant difference between DN and DD patients in the prevalence of hypertension or hyperlipidemia (see Table 1). With the exception of obesity, individuals in the the DN cohort were most likely to have any of the comorbid diagnoses examined, while patients diagnosed with diabetes and comorbid depression were significantly more likely to be diagnosed as obese.

Generally, the three most commonly prescribed antidiabetic medications were metformin, glipizide, and glyburide. These medications were three of the four most commonly prescribed medications for DN patients, with insulin lispro being the second most prescribed medication (see Table 2). Considering classes of medications, sulfonylureas and meglitinides were most commonly prescribed in all three cohorts. Generally, individuals in the DN or DD cohorts were more likely to receive an antidiabetic medication than those in the DM cohort and there were little differences in prescribing of antidiabetic medications between the DN and DD cohorts.

**Table 2:** Frequency of Use of Anti-Diabetic Medications

**Anti-Diabetic Medications**	**Diabetes (N = 55.972)**		**Diabetic Neuropathy (N = 2,146)**		**Diabetes and Depression (N = 2,379)**		**P-Value**		
	
	**N**	**%**	**N**	**%**	**N**	**%**	**DM v DN**	**DM v DD**	**DN v DD**
Sulfonylurea									
Glimepiried	2,821	5.04	180	8.29	192	8.07	*	*	0.8823
Glipizide	9,034	16.14	458	21.34	554	23.29	*	*	0.1846
Glyburide	7,446	13.30	451	21.02	466	19.59	*	*	0.3454
Any Sulfonylurea	18,207	32.53	924	43.06	1,072	45.06	*	*	0.3253
Meglitinide									
Nateglinide	222	0.40	19	0.89	6	0.25	***	0.5252	***
Repaglinide	863	1.54	63	2.94	1	2.27	*	***	0.1790
Any Meglitinide	1,078	1.93	82	3.32	60	2.52	*	0.1059	***
Biguanide									
Metformin HCL	15,744	28.13	952	44.36	1,072	45.06	*	*	0.8625
Thiazolidinedione									
Pioglitazone HCL	2,371	4.24	227	10.58	233	9.79	*	*	0.4238
Rosiglitazone Maleate	3,073	5.49	287	13.37	292	12.27	*	*	0.2650
Any Thiazolidinedione	5,283	9.44	491	22.88	494	20.77	*	*	***
Alpha Glucose Inhibitor									
Acarbose	256	0.46	13	0.61	18	0.76	0.5883	0.0939	0.7412
Miglitol	112	0.20	13	0.61	1	0.04	**	0.2222	*
Any Alpha Glucose Inhibitor	363	0.65	26	1.21	19	0.80	***	0.6553	0.2072
Insulin									
Insulin Glargine	272	0.49	68	3.17	75	3.15	*	*	0.9976
Insulin Lispro	2,837	5.07	469	21.85	359	15.09	*	*	*
Any Insulin	2,940	5.25	493	22.97	381	16.02	*	*	*

*P-value ≤ 0.0001; **P-value ≤ 0.001; ***P-value ≤ 0.05.

P values based on Tukey-Kramer method to adjust for multiple comparisons.

Not only were there differences in the cohorts with regards to use of antidiabetic medications, but there were also differences in the use of pain medications (see Table 3). The DD cohort used the most antidepressants; however, antidepressant use among the other cohorts was also non-trivial, with 10.68% of the DM cohort and 41.33% of the DN cohort using an antidepressant. A comorbid diagnosis of depression or diabetic neuropathy was associated with a significantly higher use of antidepressants, anticonvulsants, skeletal-muscle relaxants, non-narcotic pain medication, narcotics, and NSAIDs. The DD patients were significantly more likely to use a non-narcotic than the DN patients, while there were no significant differences in anticonvulsant, skeletal-muscle relaxant, narcotic, or NSAID use between the two cohorts.

**Table 3:** Frequency of Use of Pain Medications

**Pain Medications**	**Diabetes (N = 55,972)**		**Diabetic Neuropathy (N = 2,146)**		**Diabetes and Depression (N = 2,379)**		**P-Value**		
	
	**N**	**%**	**N**	**%**	**N**	**%**	**DM v DN**	**DM v DD**	**DN v DD**
Antidepressants									
SSRI	3,376	6.03	536	24.98	1,346	56.58	*	*	*
Tricyclic	1,884	3.37	366	17.05	354	14.88	*	*	**
Venlafaxine	271	0.48	58	2.70	216	9.08	*	*	*
Bupropion	750	1.34	109	5.08	333	14.00	*	*	*
Other Antidepressant	641	1.15	127	5.92	414	17.40	*	*	*
Any Antidepressant	5,979	10.68	887	41.33	1,715	72.09	*	*	*
Anticonvulsant									
Gabapentin	867	1.55	286	13.33	197	8.28	*	*	*
Other Anticonvulsant	911	1.63	137	6.38	273	11.48	*	*	*
Any Anticonvulsant	1,669	2.98	388	18.08	414	17.40	*	*	0.4693
Skeletal-Muscle Relaxants	3,623	6.47	349	16.26	404	16.98	*	*	0.6165
Non-Narcotics	1,018	1.82	88	4.10	134	5.63	*	*	**
Narcotics									
Oxycodone	2,789	4.98	370	17.24	353	14.84	*	*	***
Tramadol	1,043	1.86	129	6.01	144	6.05	*	*	0.9949
Codeine	3,128	5.59	300	13.98	306	12.86	*	*	0.2611
Hydrocodone-Acetaminophen	5,545	9.91	528	24.60	645	27.11	*	*	***
Proxyphene	3,374	6.03	350	16.31	369	15.51	*	*	0.5303
Other Narcotics	1,051	1.88	121	5.64	150	6.31	*	*	0.2740
Any Narcotics	12,383	22.12	1,065	44.63	1,152	48.42	*	*	0.6036
NSAIDs									
Cox-2	4,445	7.94	355	16.54	407	17.11	*	*	0.7753
Other NSAID	10,005	17.88	761	35.46	823	34.59	*	*	0.7367
Any NSAID	12,839	22.94	938	43.71	1,040	43.72	*	*	1.00

*P-value ≤ 0.0001; **P-value ≤ 0.001; ***P-value ≤ 0.05

P values based on Tukey-Kramer method to adjust for multiple comparisons.

Patients diagnosed with DM, DN, or DD had significant differences in total medical costs (see Table 4). Patients in both the DN and DD cohorts had significantly higher mean total medical costs than DM patients ($24,765 vs. $4,819, $19,398 vs. $4,819, p < 0.0001). This increase in costs associated with a diagnosis of neuropathy included all cost components: inpatient ($7,282 vs. $1,005, p-value < 0.0001), outpatient ($14,137 vs. $2,548, p-value < 0.0001), emergency room ($889 vs. $178, p-value < 0.0001), and pharmaceutical ($6,526 vs. $1,098, p-value < 0.0001) costs. Similarly, all cost components were also higher in the DD cohort relative to the DM cohort. A comparison of the DN and DD cohorts revealed that there were significantly higher inpatient ($4,167 vs. $3,011, p < 0.01) and outpatient ($8,862 vs. $8,109, p < 0.05) costs in the DN cohort, and no significant difference in total direct medical costs, emergency room costs or pharmaceutical costs.

**Table 4:** Medical Costs

**Diabetic Neuropathy (DN) Cohort Compared to Diabetes (DM) Cohort**		
**Cost Component**	**Comparative Costs**	**P Value**

Total Direct Medical Costs	$4819.29 v $24,764.91	*
Inpatient Costs	$1,004.78 v $7,282.16	*
Outpatient Costs	$2,548.09 v $14,137.98	*
ER Costs	$178.28 v $889.25	*
Pharmaceutical Costs	$1,098.28 v $5,622.53	*
Diabetes-Related Total Direct Medical Costs	$1,296.79 v $5,125.10	*

**Diabetes and Depression (DD) Cohort Compared to Diabetes (DM) Cohort**		

**Cost Component**	**Comparative Costs**	**P Value**

Total Direct Medical Costs	$4,819.29 v $19,397.59	*
Inpatient Costs	$1,004.78 v $3,644.51	*
Outpatient Costs	$2,548.09 v $10,895.46	*
ER Costs	$178.28 v $833.85	*
Pharmaceutical Costs	$1,098.28 v $6,526.86	*
Diabetes-Related Total Direct Medical Costs	$1,296.79 v $3,263.81	*

**Diabetic Neuropathy (DN) Cohort Compared to Diabetes and Depressed (DD) Cohort**		

**Cost Component**	**Comparative Costs**	**P Value**

Total Direct Medical Costs	---	NS
Inpatient Costs	$3,011.01 v $4,167.12	**
Outpatient Costs	$8,109.09 v $8,861.53	***
ER Costs	---	NS
Pharmaceutical Costs	---	NS
Diabetes-Related Total Direct Medical Costs	$2,783.23 v $3,455.54	*

Results from ordinary least squares, stepwise regressions controlling for age, sex, region, insurance type, and comorbidities (hypertension, hyperlipidemia, cardiovascular disease, ischemic heart disease, and obesity) using log of costs as the dependent variable.

*P-value ≤ 0.0001; **P-value ≤ 0.001; ***P-value ≤ 0.05

Patients diagnosed with DM had a mean total diabetes related medical cost of $1,297; however, with the addition of a neuropathy complication or comorbid diagnosis of depression, the mean total diabetes related costs were significantly increased, to $5,125 (p-value < 0.0001) for DN patients and $3,263 (p-value < 0.0001) for DD patients (see Table 4). The DN cohort also had significantly higher diabetes related medical costs compared to the DD cohort ($3,456 vs. $2,783, p-value < 0.0001). As a test of the robustness of the results, the multivariate cost regressions were reestimated with a 10% trim of the data in order to minimize the potential impact of outliers. General results were not sensitive to this alternative specification.

The analysis also briefly examined a small cohort of 683 diabetic patients diagnosed with both diabetic neuropathy and depression. The cohort consisted of mostly females (60%) and had a mean age of 49 years. The most common antidiabetic medications for patients with neuropathy and depression were metformin (59.44%), insulin lispro (36.16%), glyburide (29.43%) and glipizide (26.65%%). In addition to the antidiabetic medications, 77% received a prescription for narcotic medications, 61% for NSAIDs, and 34% for anticonvulsants. Total costs for individuals diagnosed with diabetic neuropathy and depression were over twice as much for those in the DN cohort ($48,281 vs. $18,665, p < 0.0001) and triple the direct medical costs for individuals with DD ($47,214 vs. $14,785, p < 0.0001).

## Discussion

Results from this study indicated significant differences in demographic characteristics, comorbidities, and medication use among individuals diagnosed with DM, DN, or DD. An examination of demographic characteristics of individuals in the DM, DN, and DD cohorts revealed that the DD cohort was most likely to be female, most likely to reside in the Midwest, and least likely to be self-insured. These findings are consistent with previous research that has found an increased prevalence of depression in females [22,23]. An examination of patient characteristics also revealed that individuals in the DN cohort, in most cases, were most likely of the three cohorts to be diagnosed with complications or comorbidities; therefore, the DN patients may be complicated to treat, and special attention may need to be paid to possible medication interactions.

The study cohorts used a variety of antidiabetic medications. For all cohorts, the most commonly prescribed antidiabetic medication was metformin and the second-generation sulfonylureas glyburide and glipizide were also frequently prescribed. While previous research has found that metformin is associated with decreased mortality [24,25], reductions in any diabetes-related endpoint [25], and improved glycemic control [26], other research has found that compared to sulfonylureas, metformin is not as cost-effective [27,28]. In contrast, a study of the Veteran's Administration found that in 1998 and 2000 the second-generation sulfonylureas were most commonly prescribed, although the percentage of users was decreasing over time [29]. There was relatively little use of insulin by individuals in the DM cohort with only 5% of the DM cohort using any insulin. In contrast, 23% of the DN cohort and 16% of the DD cohort received insulin during the twelve month post-period. While the use of insulin was less, on average, than found in the Department of Veteran Affairs (VA) study, this population was younger and more predominantly female than the VA population [29].

In addition to an examination of the antidiabetic medications used by individuals diagnosed with DM, DN, or DD, pharmacological treatments that are often used to treat painful physical symptoms were also examined. These medications, which include antidepressants, anticonvulsants, skeletal-muscle relaxants, non-narcotics, narcotics, and NSAIDs have been examined previously in the use of the management of neuropathic pain [30]. Of course, antidepressants may also be used independently for the treatment of depression that may be comorbid with diabetes. Among all cohorts, SSRIs were the most commonly prescribed class of antidepressant, although results also indicated that individuals in the DD cohort were more likely to use SSRIs, while those with DN were more likely to use tricyclics. This finding is consistent with the evidence that SSRIs are well suited for the treatment of depression [31-33] and that tricyclics may be more effective at treating neuropathic pain [30] than SSRIs. Given the painful symptoms that may accompany diabetic neuropathy, it is not surprising that the DN cohort used significantly more pain medications than the DM cohort. The DD cohort also used significantly more of all classes of pain medications than the DM cohort and significantly more antidepressants than the DN cohort. This finding complements previous research that has found significantly higher prescription medication expenditures for individuals diagnosed with DD compared to a DM cohort [34], by illustrating some significant differences in the use of specific medications between the two groups.

The difference in patient characteristics, use of antidiabetic medications, and use of pain medications between the three cohorts translated into significant differences in medical costs. Individuals diagnosed with DD had significantly higher total medical costs related to a diagnosis of diabetes, as well as higher total medical costs. These higher total medical costs consisted of significantly higher inpatient, outpatient, emergency room, and pharmaceutical costs for individuals diagnosed with DD. Prior research has found that individuals diagnosed with diabetes and comorbid depression are less likely to exercise, maintain a healthy diet, or adhere to oral hypoglycemic medication [35]. Given these outcomes associated with DD it is not surprising that this research, as well as other research, has found higher costs associated with comorbid depression [18,34].

Individuals diagnosed with DN had significantly higher costs than individuals diagnosed with DM, with total direct medical costs for DN more than five times higher than costs associated with DM. These costs are consistent with previous research, which has found significantly higher costs associated with diabetic complications [36-38]. In addition to the higher total medical costs associated with DN, individuals in the DN cohort also had significantly higher diabetes-related costs compared to individuals diagnosed with DM or DD. For example, individuals with DN had 25% higher diabetes-related costs than those diagnosed with DD. Previous research has also found that being diagnosed with neuropathy was linked to significantly higher diabetes related costs [39]. While little research has directly compared the costs associated with DN to the costs associated with DD, this study generally found that resource utilization and medical costs associated with DN were significantly higher than those associated with DD.

Prior research has examined the impact of diabetes by comparing costs for individuals with and without diabetes. Some studies have examined the impact of diabetes on society by comparing total direct and indirect healthcare costs associated with a diabetes diagnosis [40,41]. Other studies have examined the impact of diabetes on employers by examining incremental productivity costs as well as medical costs of the disease [42]. Prior research has also examined the impact of diabetic complications and comorbid diagnoses in the aggregate, but without narrowing the analysis to any specific complication or set of comorbidities [3,43]. For example, such research has produced estimates of the costs associated with chronic diabetic complications in the U.S. that range from $12 to $18 billion dollars.

In addition to examining the economic impact of diabetes, previous research has also examined the economic impact of diabetes with comorbid depression and diabetic neuropathy. For example, prior research has compared healthcare costs associated with a diagnosis of diabetes to costs for individuals with diabetes and comorbid depression and found that patients with depression had higher total healthcare costs than individuals diagnosed with diabetes and no comorbid depression [34,44,45]. While research has consistently found higher costs associated with comorbid diagnosis of depression, estimates of the amount of increased costs have varied widely. For example, the range of the increased costs for a comorbid diagnosis of depression ranges from 65% [44] to 350% [34]. Similarly, research has also shown significantly higher medical costs associated with diabetic neuropathy, with prior research estimating that among all patients diagnosed with diabetes, diabetic neuropathy is associated with 17% [46] to 27% [39] of the direct medical costs of diabetes.

One advantage of this current research is that the use of pairwise comparions allows for an examination of the relative impact of diabetic neuropathy or diabetes with comorbid depression compared to a diagnosis of diabetes. As such, the research provides additional evidence concerning the relative impact that comorbid depression or diabetic neuropathy has on resource utilization in general and medical costs in particular. In addition, the analyses also compared the relative impact of diabetes and depression to diabetic neuropathy and briefly examined the incremental costs associated with individuals who have been diagnosed with both diabetic neuropathy and comorbid depression. This research also allows for a more thorough understanding of resource utilization by looking at the use of antidiabetic medications, commonly prescribed pain medications, as well as direct medical costs, major components of direct medical costs, and diabetes-related direct medical costs.

The findings of this study should be interpreted in the context of the limitations of the study design. First, this study was conducted using an administrative claims database and included only patients with medical and prescription benefit coverage; therefore, the results may not be generalizeable to other populations. Second, the use of diagnostic codes to identify individuals is not as rigorous as formal diagnostic assessments for identifying people with diabetic neuropathy or depression. In addition, the database used in this study did not allow for a categorization of primary or secondary diagnoses. Therefore, diabetes- related total direct medical costs were constructed from any diagnoses of diabetes. While this methodology may likely bias the estimates of diabetes related costs upwards, there is no reason to believe that it would bias the relative difference in diabetes-related costs among the DM, DN and DD cohorts. Third, the use of medical claims data precludes the use of patient assessments; as a result, the analysis cannot examine quality of life, functioning, or any clinical outcomes. Finally, the analyses focused exclusively on resource utilization and direct medical costs associated with a diagnosis of diabetes, diabetes with neuropathy, and diabetes with comorbid depression; therefore, it did not include any other potentially important costs, such as productivity costs and caregiver burden.

## Conclusion

In conclusion, this retrospective database study compared patient characteristics, medication use, and direct medical costs in diabetic patients with and without complications of neuropathy and comorbid depression. There were significant differences in comorbidities and medication use among the three study cohorts. These differences translated into significantly higher direct medical costs for individuals diagnosed with a diabetic complication of neuropathy or with comorbid depression.

## Authors' contributions

KL and SA were responsible for conceived the study, participated in the study design, participated in the interpretation of results, and helped draft the manuscript. ML participated in the study design, performed statistical analyses, participated in the interpretation of results, and drafted the manuscript.
